# Determining Gaps in Publicly Shared SARS-CoV-2 Genomic Surveillance Data by Analysis of Global Submissions 

**DOI:** 10.3201/eid2813.220780

**Published:** 2022-12

**Authors:** Elizabeth C. Ohlsen, Anthony W. Hawksworth, Kimberly Wong, Sarah Anne J. Guagliardo, James A. Fuller, Michelle L. Sloan, Kevin O’Laughlin

**Affiliations:** Centers for Disease Control and Prevention, Atlanta, Georgia, USA

**Keywords:** SARS-CoV-2, viral surveillance, pandemic, COVID-19, coronavirus disease, severe acute respiratory syndrome coronavirus 2, viruses, respiratory infections, zoonoses, vaccine-preventable diseases

## Abstract

Viral genomic surveillance has been a critical source of information during the COVID-19 pandemic, but publicly available data can be sparse, concentrated in wealthy countries, and often made public weeks or months after collection. We used publicly available viral genomic surveillance data submitted to GISAID and GenBank to examine sequencing coverage and lag time to submission during 2020–2021. We compared publicly submitted sequences by country with reported infection rates and population and also examined data based on country-level World Bank income status and World Health Organization region. We found that as global capacity for viral genomic surveillance increased, international disparities in sequencing capacity and timeliness persisted along economic lines. Our analysis suggests that increasing viral genomic surveillance coverage worldwide and decreasing turnaround times could improve timely availability of sequencing data to inform public health action.

Viral genomic surveillance is a critical source of information for understanding and responding to the COVID-19 pandemic. Continued high levels of transmission of SARS-CoV-2 worldwide afford myriad opportunities for natural selection; selection pressures favor viral strains with such traits as faster transmission and increased immune escape (*1*). Emerging strains are designated variants of interest and variants of concern (VOCs) by the World Health Organization (WHO) if they have heightened public health or clinical importance because of increased transmissibility, immune escape, increased clinical severity, or other factors (*2*). Efforts are needed to monitor emerging strains of the SARS-CoV-2 virus and identify and classify variants to guide public health response and to aid in the development of diagnostic tests, therapeutics, and vaccines (*3*). As of March 21, 2022, more than 9.4 million SARS-CoV-2 sequences had been uploaded to the GISAID database (https://www.gisaid.org), the leading public online repository for viral genomic data; nearly 4 million SARS-CoV-2 sequences were uploaded to GenBank (https://www.ncbi.nlm.nih.gov/genbank) by that date. The importance of improving viral genomic surveillance capacity around the world is recognized through many initiatives aiming to do so, including through the WHO (*4*) and other international partnerships (*5*). Despite this continued effort, previous analyses have found heterogeneity in publicly available sequencing coverage across regions and countries, with substantial disparities between high-income and low- and middle-income countries (*6*–*8*; A.F. Brito et al., unpub. data, https://doi.org/10.1101/2021.08.21.21262393). Highlighting and understanding these disparities is important because VOCs can emerge from any part of the world, including places where sequencing capacity is low. 

A recent case study illustrated the benefits to local and global communities that occurred after publication of South Africa viral genomic surveillance data (*9*). Those benefits included more opportunities for South Africa researchers to collaborate on an international level, better international collaboration around COVID-19 prevention and vaccination in Africa, and improved insights into SARS-CoV-2 transmission in South Africa, which informed public health policy. We present an analysis that aims to update the progress of sequencing capacity up through the emergence of Omicron as a VOC, including the number of sequences and timely sharing of the results, to better understand where further support is needed. Our analysis of publicly available viral genomic surveillance data considers the impact of the timeliness of such data to inform major international public health actions during early variant emergence. To expand findings from previous analyses of publicly available viral genomic sequencing data that demonstrated socioeconomic inequalities in viral genomic surveillance coverage (*7*; A.F. Brito et al., unpub. data), we examined the rapid expansion of viral genomic surveillance from the emergence of Omicron and included time-to-submission of collected sequences to assess timeliness. Our analysis further supports the conclusion that addressing these inequalities would improve global pandemic response and preparedness.

## Methods

### Data

The GISAID database and GenBank are public databases containing genomic sequencing data voluntarily submitted by laboratories worldwide. All available SARS-CoV-2 sequence metadata associated with human infections were downloaded from the GISAID and GenBank Web sites. We obtained reported data on SARS-CoV-2 infections by week from Our World in Data (https://ourworldindata.org) (*10*) and by population from the World Bank (https://databank.worldbank.org/home.aspx) (*11*) for countries and territories.

### Inclusion Criteria and Data Management

As of March 21, 2022, there were 9,409,674 sequences (7,280,739 with complete collection and submission dates) from 209 countries and territories in GISAID and 3,967,425 sequences (2,289,627 with complete collection and submission dates) from 111 countries in GenBank; the earliest available sequence collection date was January 1, 2020. For our analysis, we removed duplicate sequences (those with identical sequence and metadata that were uploaded to both databases). We extracted variables from metadata that included specimen collection date, submission date, and country or territory of collection (hereafter country); we excluded sequences lacking that information from our analysis, including any sequence containing incomplete information for month, day, or year of collection. We used a local instance of the computational tool PANGOLIN version 3.1.20 to obtain variant information (Pango lineage) from sequences. We also excluded sequences designated as Omicron with collection dates before the internationally recognized first detection of the Omicron variant (*12*) (10 were collected before November 8, 2021). We included all sequences designated Alpha or Delta if they met other inclusion criteria; <100 Alpha sequences appeared in the dataset before October 2020 and <100 Delta sequences appeared in the dataset before December 2020. We excluded sequences from countries lacking a WHO region designation (listed at https://www.who.int/countries) or lacking a World Bank income designation (available at https://datahelpdesk.worldbank.org).

We assumed the sequence submission date to reflect the first date a sequence was made publicly available, and we then calculated the lag time elapsed between the collection date and submission date. We analyzed the proportion of sequences that featured an elapsed time between collection and submission of <14 days because this threshold represents the 99th percentile of the duration of wild-type SARS-CoV-2 incubation time (*13*), an important metric to inform public health case investigations.

We selected different periods of time during the pandemic to highlight important differences between countries. To compare sequencing capacity over a time period when most countries had sustained community transmission and had established testing programs, the 2021 subset includes sequences from specimens collected during the year 2021. To avoid lag time artifact, we included only sequences collected before January 1, 2022, in this subset because, based on median lag times, most samples collected during 2021 would have been submitted by the date of data retrieval in March 2022.

We chose three 8-week time periods that approximately correspond to the first global waves of the Alpha (December 6, 2020–January 30, 2021), Delta (June 6–July 31, 2021), and Omicron (December 6, 2021–January 30, 2022) VOCs. We used the dates of major international public health actions, such as recognition of VOCs or implementation of international travel restrictions, to contextualize the number of sequences submitted and the number of sequences collected by these dates.

### Analysis

We used descriptive statistics to analyze the number of sequences submitted to GISAID and GenBank by country, WHO region, and World Bank income designation; sequences submitted within 14 days of collection were analyzed also. We report results by total sequences, by sequences per million population, and by sequences per 100,000 reported SARS-CoV-2 infections. We compared per capita and per infection metrics to identify differences that could be influenced by varying test availability in different countries.

We performed additional similar descriptive analysis on the 2021 subset and the 3 time periods of VOC global emergence. We used χ^2^ tests of homogeneity to test the null hypothesis that the distribution of sequences was similar by World Bank income category and WHO region, and we used Kruskal-Wallis tests to evaluate the null hypothesis that the median number of uploaded sequences were similar by World Bank income category across the 3 selected 8-week periods; we considered p values <0.05 significant. We reported the number of VOC sequences collected and the number of VOC sequences submitted around the time of international public health actions introduced to mitigate the spread of that VOC in context of those dates.

### Ethics Considerations

This activity was reviewed by Centers for Disease Control and Prevention (CDC). The analysis was conducted according to applicable federal law and CDC policy (45 C.F.R. part 46.102(l)(2), 21 C.F.R. part 56; 42 U.S.C. Sect.241(d); 5 U.S.C.0 Sect.552a; 44 U.S.C. Sect. 3501 et seq).

## Results

### Descriptive Statistics

After removing duplicate sequences (433,703), sequences with incomplete dates (3,806,733), and sequences without both a World Bank income designation and a WHO region designation (21,593), a total of 9,115,070 sequences were available for analysis. The mean number of sequences per country/territory was 48,744 (median 1,006, interquartile range 218–10,570). Of the total sequences analyzed, 6,533,870 (71.7%) were collected during January 1–December 31, 2021, and are included in the 2021 subset (Table 1).

**Table 1:** Sequencing volume by population and detected SARS-CoV-2 infections and submission lag, World Bank income category, and WHO regions based on data from GISAID and GenBank, 2021*

Category	No. countries	Total no. (%) sequences	Sequences/ 1 million population	Sequences/100,000 SARS-CoV-2 reported infections	Median lag time, d (IQR)	
World Bank income category						
Low	24	6,612 (0.1)	11	524	98 (61–148)
Lower middle	43	172,582 (2.6)	52	352	71 (41–115)
Upper middle	50	350,309 (5.4)	137	556	34 (19–65)
High	68	6,004,367 (91.9)	**5,040**	**5,547**	20 (11–35)
WHO Regional Office						
Africa	41	54,115 (0.8)	47	987	55 (32–101)
The Americas	42	2,617,580 (40.1)	2,611	3,512	27 (18–47)
United States	1	2,161,680 (82.6)	6,493	5,477	24 (17–39)	
Non–United States	41	455,900 (17.4)	154	1,302	42 (28–84)	
Eastern Mediterranean	20	12,264 (0.2)	17	101	56 (21–135)	
Europe	54	3,433,142 (52.5)	3,767	4,066	14 (9–25)	
United Kingdom	1	1,542,137 (45.9)	25,200	14,505	10 (8–14)	
Non–United Kingdom	53	1,891,005 (55.1)	1,767	2,362	20 (13–34)	
South-East Asia	9	139,846 (2.1)	138	818	63 (37–108)	
Western Pacific	19	276,923 (4.2)	**72**	**1,259**	49 (29–72)	

*A total of 6,533,870 sequences were collected in 2021. Bold indicates significance (p<0.001 by χ^2^ test). GISAID, https://www.gisaid.org; IQR, interquartile range; WHO, World Health Organization.

### Comparisons by Income Category

**Figure 1:**
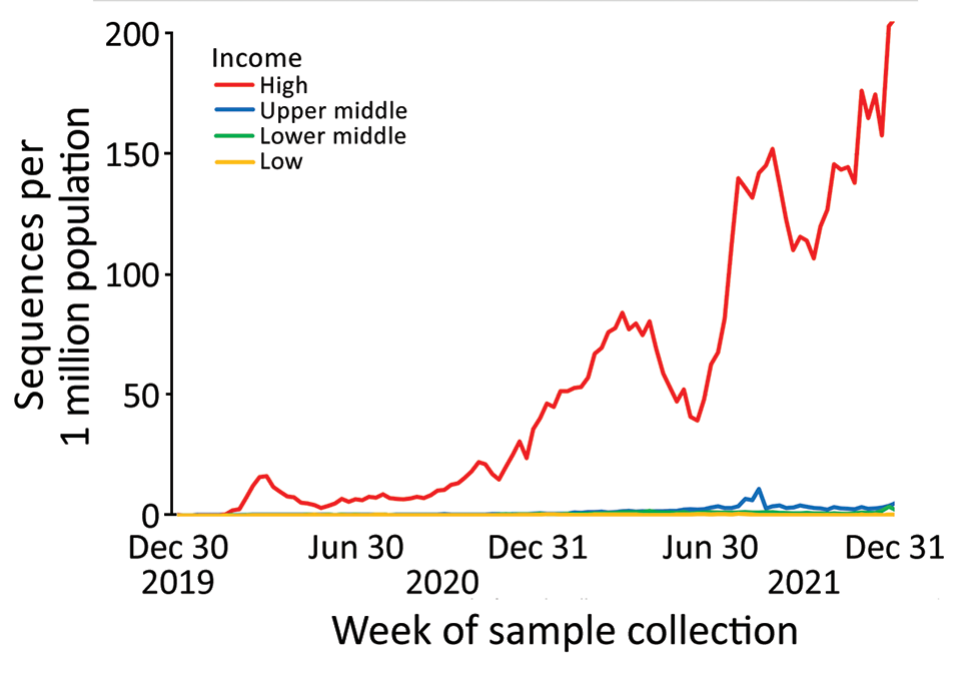
Weekly volume of SARS-CoV-2 sequences collected per 1 million population by income category of country, GISAID (https://www.gisaid.org) and GenBank, 2020–2021. Data include only populations of countries submitting >1 sequences. Data are truncated at the end of 2021 to avoid lag time artifact impacting comparison of sequencing volume nearer to the date of data access on March 21, 2022, because only collected samples that were also submitted by March 21, 2022, appear in these data.

**Figure 2:**
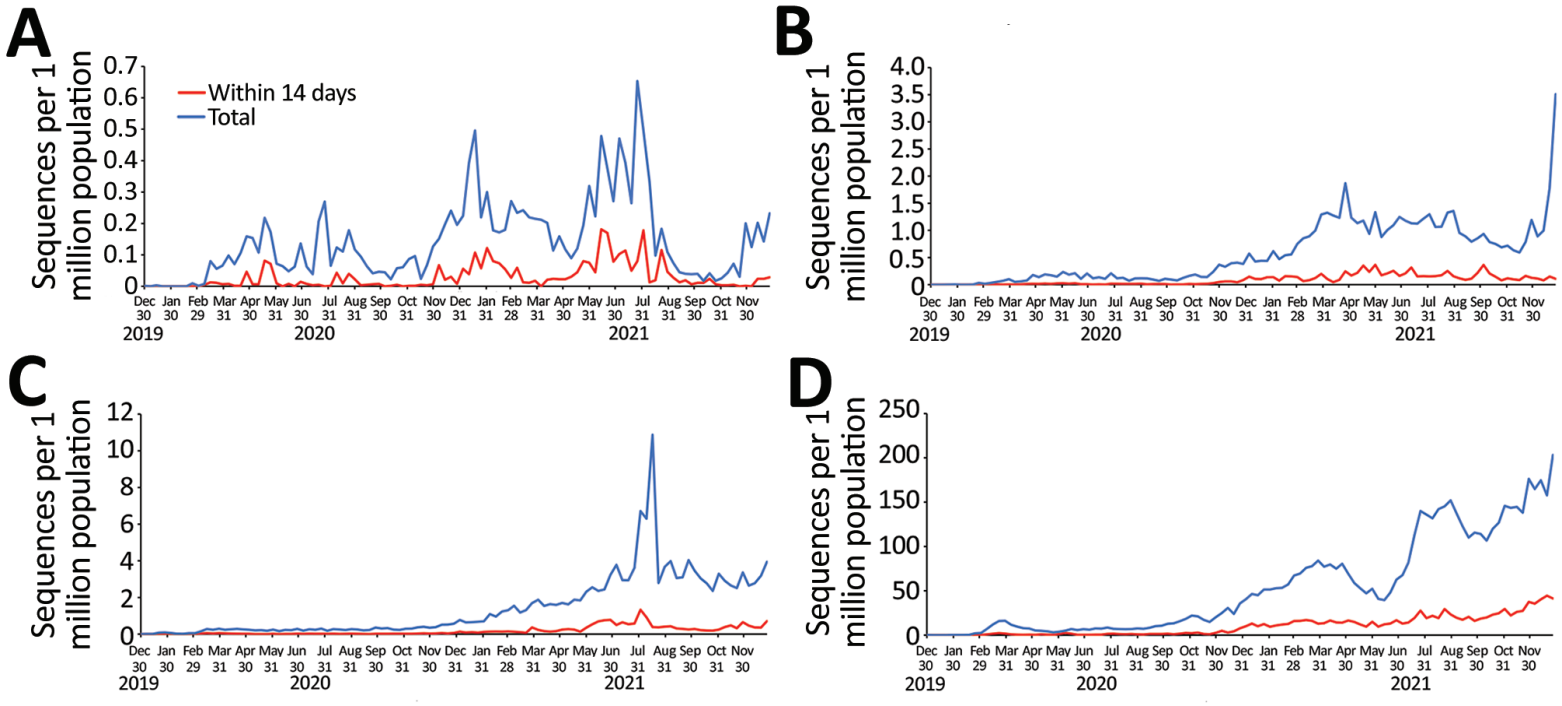
Total SARS-CoV-2 sequences and sequences submitted within 14 days of collection, by population and income category, GISAID (https://www.gisaid.org) and GenBank, 2020–2021. A) Low-income countries; B) lower-middle-income countries; C) upper-middle-income countries; D) high-income countries. Dates indicate sequence collection dates. Data include only populations of countries submitting >1 sequence.

During 2020 and 2021, high-income countries had the greatest number of submissions per capita and increased average daily submissions by >10 times any other income category (Figure 1). Sequences submitted within 14 days of collection increased in all World Bank income categories for this period but remained a minority of sequences submitted during that time in each category (Figure 2).

In the 2021 subset, high-income countries submitted 456 times more SARS-CoV-2 sequences than low-income countries when adjusting for population (5,040 sequences/1 million population vs. 11 sequences/1 million population; p<0.001) and 36 times more than upper-middle-income countries (5,040 sequences/1 million population vs. 137 sequences/1 million population; p<0.001) (Table 1). Low-income countries had a higher proportion of sequences submitted per reported SARS-CoV-2 infection than lower-middle-income countries (Table 1) but a lower proportion than upper-middle-income or high-income countries. High-income countries had the shortest median lag time in sequence submission, 20 days, whereas low-income countries had the longest median lag time, 98 days.

### Comparisons by WHO Region

In the 2021 subset, the WHO Regional Offices for the Americas and Europe had the most sequences per capita and per infection and had the shortest lag times; the Eastern Mediterranean region had the lowest sequences per capita and per infection and had the longest lag times (Table 1). A substantial number of submissions from the Americas and European regions were from the United States (82%) and the United Kingdom (50%) (Table 1). By region, the Eastern Mediterranean region had the least sequencing relative to both population and reported infections; differences among regions were significant after accounting for population (p<0.001). The Regional Office for Africa had more reported sequences relative to infections detected than the South-East Asia region, but the South-East Asia region had somewhat higher sequencing coverage per capita. Lag times decreased as per capita sequencing volume increased by region (Table 1).

Sequencing volume from some countries and territories was low; for 29 countries and territories, <100 total sequences were submitted. Across these countries and regions with a relatively low submission of sequences, each World Bank income category was represented, including 10% of high-income countries (7/69), 14% of upper-middle-income countries (7/51), 14% of lower-middle-income countries (6/43), and 38% of low-income countries (9/24). By WHO region, 17% of countries or territories from the Africa region (7/41) submitted <100 sequences in total, as did 21% from the region of the Americas (9/43), 20% from the Eastern Mediterranean region (4/20), 7% from the Europe region (4/54), and none from the South-East Asia region (0/9). Of countries or territories from the Western Pacific region, 25% (5/20) submitted <100 sequences in total (data not shown).

### Comparisons across Alpha, Delta, and Omicron Emergence

Submitted sequences per 1 million population more than doubled between the selected months that marked the global emergence of the Alpha variant (49 sequences/1 million population) and those months that distinguished the emergence of the Delta variant (100 sequences/1 million population). Submitted sequences per 1 million population again more than doubled between the months distinguishing the Delta variant and those that marked the emergence of Omicron (243 sequences/1 million population) (Table 2). When reported infections are accounted for, a similar increase is seen between Alpha (981 sequences submitted/100,000 reported infections), Delta (2,017/100,000 reported infections), and Omicron (4,890/100,000 reported infections) (Table 2). Sequences submitted within 14 days of collection doubled between Alpha and Delta periods, from 10.5 to 21.5/1 million population, and doubled again between Delta and Omicron, to 46.7/1 million population, illustrating a global growth in viral genomic surveillance capacity with later variants (Table 2). 

**Table 2:** SARS-CoV-2 sequences submitted to GISAID and GenBank with collection dates during 8-week periods of initial global transmission waves of Alpha, Delta, and Omicron variants of concern*

Category	Alpha, 2020 Dec 6–2021 Jan 30	Delta, 2021 June 6–Jul 31	Omicron, 2021 Dec 6–2022 Jan 30	p value†
Sequences collected	376,637	774,534	1,877,225	
Countries submitting sequences	168	164	151	
Median lag time, d	39	23	17*	
Mean sequences submitted/1 million population	48.8	100.4	243.3	<0.001
Low income	2.1	3.2	1.1	
Lower-middle income	3.4	8.7	13.6	
Upper-middle income	4.7	23.7	28.3	
High income	295.6	573.2	1,476.1	
Mean sequences/100,000 SARS-CoV-2 reported infections	981.0	2,017.4	4,889.6	<0.001
Low income	795.8	655.1	170.4	
Lower-middle income	302.1	319.2	378.3	
Upper-middle income	115.8	493.2	351.4	
High income	1,457.2	8,899.4	2,074.2	
Sequences collected within 14 d lag time (% total as of 2022 Mar 20)	81,358 (21.6)	165,758 (21.4)	360,022 (19.2)	
Countries submitting sequences within 14 d of sample collection	118	115	94	
Mean sequences submitted/1 million population within 14 d of collection	10.5	21.5	46.7	<0.001
Low income	0.4	0.8	0.08	
Lower-middle income	0.7	1.6	1.1	
Upper-middle income	0.6	4.9	3.5	
High income	64.7	123.5	291.6	

*Mean time during Omicron cannot be accurately compared to mean lag time during Alpha or Delta because data from GISAID and GenBank were retrieved <2 months after the end of the Omicron period examined in this analysis. GISAID, https://www.gisaid.org. †By Kruskal-Wallis test.

When examined by World Bank income category, high-income countries had both the highest growth and the highest overall sequencing total, by population and by reported infections. Other income categories displayed diminished or even no growth in these measures between the Delta and Omicron periods (Table 2). For sequences submitted within 14 days of collection, high-income countries nearly doubled sequencing submissions between each period: 65/1 million population during Alpha, 124/1 million population during Delta, and 292/1 million population during Omicron. During the same time, low-, lower-middle-, and upper-middle-income countries doubled sequences submitted within 14 days of collection between the Alpha and Delta periods but had fewer during Omicron than Delta. For example, lower-middle-income countries submitted 0.7 sequences/1 million population within 14 days of collection during Alpha and 1.6/1 million population during Delta but just 1.1/1 million population during Omicron (Table 2).

### Availability of Surveillance Data to Inform Public Health Action

On December 18, 2020, WHO designated the Alpha variant a VOC (*2*), and on December 19, 2020, at least 7 countries implemented specific travel restrictions aimed to slow transmission of Alpha (*14*). Based on data pulled from the 2 public databases, 11,586 Alpha sequences were collected before December 19, but only 1,872 (16%) of those had been submitted by December 19 (Table 3). On May 10, 2021, the date WHO designated Delta a VOC (*2*), 25,433 Delta sequences from 104 countries on 6 continents had been collected but, of those, just 1,910 sequences (8%) had been publicly submitted. Similarly, on November 26, 2021, the date when WHO designated Omicron a VOC (*2*) and at least 23 countries implemented travel restrictions (*16*), 1043 Omicron samples had been collected from 38 countries on 5 continents, but only 76 sequences (5.6%) from 3 countries on 2 continents were submitted to GISAID or GenBank before that date (Table 3).

**Table 3:** Geographic distribution of Alpha, Delta, and Omicron SARS-CoV-2 sequences before dates of selected international public health actions, based on data from GISAID and GenBank, 2020–2021*

Variant	International action (date implemented)	Sequences collected before that date	Geographic diversity of origin	Sequences submitted before that date (% of total collected)	Geographic diversity of origin
Alpha	International travel restrictions (*14*) (2020 Dec 19)†	11,586	48 countries (5 continents)	1,872 (16)	4 countries (2 continents)
Delta	WHO-designated VOC (*2*) (2021 May 11)	28,257	116 countries (6 continents)	2,257 (8.0)	39 countries (5 continents)
Delta	CDC-designated VOC (*15*) (2021 Jun 15)‡	121,071	137 countries (6 continents)	46,946 (39)	66 countries (6 continents)
Omicron	WHO-designated VOC (*2*); international travel restrictions (*16*) (2021 Nov 26)	1,365	48 countries (6 continents)	76 (5.6)	3 countries (2 continents)

*CDC, United States Centers for Disease Control and Prevention; GISAID, https://www.gisaid.org; VOC, variant of concern. WHO, World Health Organization. †WHO designated Alpha a variant of concern on December 18, 2020 (*2*). ‡Several countries implemented (Germany, Hong Kong, Lithuania, Slovakia, Belgium) or extended (United States) travel restrictions due to Delta during June or July 2021.

## Discussion

The bank of global, publicly available SARS-CoV-2 genomic sequence data increased substantially during the COVID-19 pandemic. The number of sequences submitted to public databases more than doubled overall (and increased in all income categories) between the Alpha period and the Delta period, doubling again between the Delta and Omicron periods. This increase in sequence submissions might reflect the impact of technological advancements, the continued high utility of genomic sequencing data, and increased prioritization of genomic surveillance between these periods. Continuing to strengthen laboratory and data sharing infrastructure and international partnerships for viral genomic surveillance could improve monitoring and early detection of SARS-CoV-2 variants and might contribute to monitoring and detection of other pathogens.

Despite the general trend of increased sequencing during the pandemic, disparities between World Bank income categories and WHO regions increased during the Omicron wave. Similarly, the number of sequences submitted within 14 days of collection increased between the emergence of 3 major SARS-CoV-2 variants, but disparities persisted in the volume of sequences submitted within 14 days of collection along economic lines. The only exception to this trend was the finding that the lowest income category of countries had higher sequences submitted per 100,000 reported infections detected than did the lower-middle category. This difference is likely related to lower testing and case detection in the lowest income category; when examined by population, per capita sequencing was substantially lower in the lowest income category than the lower-middle category. A greater proportion of low-income countries were associated with <100 sequences compared with other income categories, which might be the result of partnerships with other countries for sequencing. Overall median lag times between sample collection and public sequence submission exceeded 14 days, reflecting a need to improve sequencing turnaround time to inform timely global public health decision-making. 

Our analysis cannot distinguish between the time from sample collection to sequence result and the time from sequence result to submission to a public database because these variables are not included in GISAID or GenBank metadata. However, using viral genomic surveillance data to inform rapid international public health action depends on both rapid sequencing and the timely sharing of data. For example, global knowledge of Omicron began with timely identification of an unusual SARS-CoV-2 lineage identified by a team of researchers in Botswana, who shared their findings with colleagues in South Africa and on public servers within days (*17*). Sequences made available long after collection can still contribute to knowledge on a pathogen’s transmission dynamics and other characteristics, so reducing the time to sequencing and encouraging prompt sharing of data could improve the quality and usefulness of information for public health action.

In terms of limitations regarding our analysis, we examined only sequences uploaded to GISAID and GenBank. Although those are the largest 2 repositories of viral genomic surveillance data, they contain only those reports that laboratories and countries choose to make public. Also, by choosing a time period comparison including a relatively recent 2-month period, the data from the Omicron period reflect only sequences submitted and available to download as of March 21, 2022, and do not include sequences that may have been collected during the Omicron period but submitted after this date. Because of this, the observed differences in the volume of submitted sequences between the Omicron period and the earlier 2 periods are likely larger than these data reflect. Finally, overall average lag times from the more recent Omicron period cannot be compared with those from the Alpha or Delta periods because the Omicron period was relatively close to the data cutoff and therefore more likely to include sequences with short lag times. The volume of sequences submitted within 14 days of collection can be compared across periods.

For many reasons, including incomplete and variable vaccination coverage (*18*), continued viral transmission is anticipated and the emergence of new SARS-CoV-2 variants is expected (*19*). The availability of samples for sequencing depends on the availability of testing. Because testing is limited in many settings (I. Bergeri et al., unpub. data, https://www.medrxiv.org/content/10.1101/2021.12.14.21267791v2), samples available for sequencing may not represent the true diversity of viral genomes within countries. Testing, viral genomic surveillance, and sharing of data are critical to early detection of new variants and accurate assessment of their spread. The unequal viral genomic surveillance highlighted by this analysis suggests a new variant can circulate widely before detection and public sharing of the new variant’s genomic information. 

The 3 VOCs we assessed were already present in many countries at the time travel restrictions were imposed. One analysis of Omicron-related travel restrictions found that most countries issuing entry bans did not modify them after widespread community transmission of Omicron was reported elsewhere, and most did not add increased testing or quarantine requirements for travelers (*20*). Faster sequencing and more timely public sequencing availability might contribute to better understanding of how widely variants have spread at the time of their designation as VOCs and might also help encourage policies supporting evidence-based transmission prevention measures, such as increasing masking (*21*), rather than reliance on travel restrictions, which might have only a modest effect on transmission, particularly after introduction has already occurred (*22*). 

Supporting the expansion of representative testing across and within countries and regions could increase the quantity of specimens available for sequencing. Addressing the global inequity of viral genomic surveillance information by supporting the expansion of representative viral genomic surveillance—particularly in low-, lower-middle-, and upper-middle-income countries, including through such efforts as the African Pathogen Genomics Initiative (*23*)—might increase the probability of early detection and characterization of new variants and timely implementation of tailored responses, like nonpharmaceutical interventions, diagnostic approaches, and vaccines. Encouraging timely public sharing of viral genomic surveillance data by supporting countries that report detection of new variants, new outbreaks, or new pathogens could help bolster the ability of all countries to publicly share surveillance information and to set effective, timely public health policy. Together, these efforts could promote global health security during this and future pandemics.
